# Refining mini-monovision with monofocal plus intraocular lenses

**DOI:** 10.1007/s00417-025-07013-2

**Published:** 2025-11-08

**Authors:** Tadas Naujokaitis, Gerd U. Auffarth, Zhiyi Wu, Fátima Cuéllar, Ramin Khoramnia, Grzegorz Łabuz

**Affiliations:** 1 Department of Ophthalmology, The David J. Apple Center for Vision Research, University of Heidelberg, INF 400, Heidelberg, 69120 Germany; 2 Department of Ophthalmology, Faculty of Medicine and University Hospital Carl Gustav Carus, TU Dresden, Fetscherstr. 74, 01307 Dresden, Germany; 3 Eye Center, The Second Affiliated Hospital, School of Medicine, Zhejiang University, Hangzhou, 310009 China; 4 Applied Optics and Image Processing Research Group, Department of Optics and Optometry, Universitat Politècnica de Catalunya BarcelonaTech, Violinista Vellsollà 37, 08222 Terrassa, Spain

**Keywords:** Mini-monovision, Monovision, Monofocal plus, Enhanced monofocal, Intraocular lens

## Abstract

**Purpose:**

To evaluate simulated mini-monovision with two enhanced monofocal intraocular lenses (IOLs).

**Methods:**

Using an optical bench, modulation and phase transfer functions of the Acunex Quantum (Teleon Surgical) and RayOne EMV (Rayner) IOLs with nominal powers of 10D, 20D, and 30D were measured. Simulated visual acuity (simVA) defocus curves were derived. Mini-monovision with an offset of -0.25D to -1.0D was simulated by shifting the simulated defocus curves and by applying quadratic summation to the acquired United States Air Force (USAF) chart images.

**Results:**

The Acunex Quantum produced a more extended depth of focus than RayOne EMV in simulated defocus curves and USAF chart analysis. Higher-powered lenses tended to provide a slightly greater depth of focus. In a simulated mini-monovision, the − 1.0D myopic offset provided the greatest depth of focus, with a simVA of 0.1 logMAR and better up to approximately − 2.0D to −2.4D with Acunex Quantum and − 1.8D to −2.1D with RayOne EMV. Higher offsets with lower IOL powers resulted in a slightly worse simulated binocular distance vision in the image analysis, more noticeable with the Acunex Quantum, and a slight reduction of binocular simVA between the refractive targets in both IOLs.

**Conclusions:**

Our findings suggest that a mini-monovision approach can be successfully applied with either lens: the Acunex Quantum potentially providing a greater depth of focus, and the RayOne EMV a slightly better low-defocus tolerance for lower IOL powers in a non-dominant eye. The differences between the lenses diminished with increasing IOL power.

**Supplementary Information:**

The online version contains supplementary material available at 10.1007/s00417-025-07013-2.

## Key messages

*****What is known?***:**

- Mini-monovision aims to increase the depth of focus in binocular vision.

- RayOne EMV is an enhanced monofocal intraocular lens, specifically designed to be used in a mini-monovision approach.

*****What is new?***:**

- Laboratory findings suggest that other enhanced monofocal intraocular lenses can be used in mini-monovision, maintaining a good distance visual acuity while improving intermediate and near vision.
- In laboratory simulations, the Acunex Quantum (Teleon Surgical) provided a greater depth of focus, while the RayOne EMV (Rayner) showed a slightly better low-defocus tolerance for lower IOL powers.
- Higher offsets resulted in a greater simulated depth of focus but had a slight negative effect on distance vision.

**Supplementary Information:**

The online version contains supplementary material available at 10.1007/s00417-025-07013-2.

## Introduction

Monovision is an approach in surgical planning in which, to increase spectacle independence, intraocular lenses (IOLs) are implanted with different refractive targets for each eye [1]. In conventional monovision using monofocal IOLs, one eye is left emmetropic, while the fellow eye is purposely set for myopia of approximately − 2.0D [2]. By creating this refractive difference (anisometropia), patients can gain depth of focus in binocular vision [1]. Although monovision is tolerated well by most patients, the marked interocular difference produced by conventional monovision may not be accepted by some [2]. Mini-monovision, also known as micro-monovision, differs from conventional monovision in that it has a reduced difference between the refractive targets, providing the patient with more similar monocular images [3].

Ocular dominance testing is conventionally performed before monovision and mini-monovision treatments, and the dominant eye is usually set for emmetropic target [1, 2]. However, recent studies have indicated that the predominant eye may be constantly changing as there is a constant rivalry between both eyes [4]. Although we refer to the dominant eye as the eye with the emmetropic target refraction, the selection of eye for the emmetropic target in the clinical practice may differ from this convention and should be discussed with the patient individually.

RayOne EMV (Rayner, Worthing, UK) is a monofocal IOL purposely designed to work in a mini-monovision configuration [5]. This lens utilizes positive spherical aberration (SA) to extend the depth of focus in the dominant eye and to mitigate the effects of myopic shift in the non-dominant eye [5]. Acunex Quantum (Teleon Surgical, Spankeren, the Netherlands) is a monofocal IOL with improved intermediate vision compared to a standard monofocal IOL [6]. This improved depth of focus could give an additional advantage when used in the mini-monovision approach. The objective of the study was to compare the simulated mini-monovision with Acunex Quantum vs. RayOne EMV IOLs, based on optical-bench measurements of the lenses.

## Materials and methods

### Intraocular lenses

The IOL models investigated in the study were the Acunex Quantum AN6Q and the RayOne EMV. A total of twelve IOLs were tested: six samples per model, two each with a nominal refractive power of 10D, 20D, and 30D.

The Acunex Quantum is a hydrophobic-acrylic IOL with a refractive index of 1.54 and an Abbe number of 41 [6]. The central area of the IOL optic features increased power, referred to by its manufacturer as the Q-zone, followed by a gradual power decrease until reaching the peripheral monofocal part [6]. The lens features an aberration-correcting design intended to lower the cornea’s primary SA.

The RayOne EMV IOL is made of a hydrophilic-acrylic material, has a refractive index of 1.458 at 35 °C and an Abbe number of 56 [5]. To increase the depth of focus, positive spherical aberration is induced by the central part of the lens optic amplifying the positive spherical aberration of the cornea, while the periphery has a monofocal aberration-neutral design [5].

### Optical metrology

We performed optical bench measurements using the OptiSpheric IOL PRO2 (Trioptics, Wedel, Germany) optical metrology device. Its illustration and detailed description can be found on the manufacturer’s website [7]. The IOL power was measured using the magnification method in monochromatic light (546 nm), according to ISO 11979-2 [8]. Both IOL models were assessed at room temperature. For the RayOne EMV, the measured IOL power was adjusted to account for the temperature difference from in situ conditions, and an additional correction was applied to address longitudinal SA, as recommended by the manufacturer.

### Image quality metrics

The optical quality measurements were performed in polychromatic light, using a spectral filter simulating the spectral sensitivity of the human eye [9] and the model cornea with 0.27 μm of SA at 5.15 mm. Aperture sizes of 3 mm and 4.5 mm at the IOL plane were used to simulate photopic and mesopic conditions. After the best focus was determined according to the through-focus modulation transfer function (MTF), the MTF and phase transfer function (PFT) measurements were performed at a defocus from + 0.50D to −2.5D at the spectacle plane. In addition, the United States Air Force (USAF) resolution test chart was recorded through the lenses at different levels of defocus.

### Simulation of visual acuity

Based on the measured MTF and PTF, we calculated the weighted optical transfer function (wOTF) as described by Alarcon et al., using the following formula:

$$\:wOTF=\frac{d}{150}\:{\sum\:}_{f=1}^{\frac{150}{d}}MTF\left(fd\right)cos\left(PTF\left(fd\right)\right){CSF\left(fd\right)}_{neural}$$

where f is the spatial frequency in line pairs per mm (lp/mm) with a resolution of d = 1 lp/mm, and the CSF refers to the neural contrast sensitivity function based on the findings by Campbell and Green [10, 11]. To obtain simulated visual acuity (simVA) in the Logarithm of the Minimum Angle of Resolution (logMAR), the wOTF^b^ (b=−0.36) was calculated.

### Simulation of mini-monovision

A binocular effect of mini-monovision was simulated by incorporating a formula predicting binocular defocus curve derived from evaluation of the two studied models using a SimVis Gekko (2EyesVision, Madrid, Spain). The results of those measurements are presented elsewhere [12]. Based on that study, where plano implantation, as well as mini-monovision with a refractive target of −0.50D and − 1.0D were simulated and the binocular effect quantified, a formula predicting the binocular defocus curve was derived:

$$\:\mathrm{B}\mathrm{i}\mathrm{n}\mathrm{o}\mathrm{c}\mathrm{u}\mathrm{l}\mathrm{a}\mathrm{r}\:\mathrm{E}\mathrm{f}\mathrm{f}\mathrm{e}\mathrm{c}\mathrm{t}=1.17-0.24\bullet\:\varDelta\:\mathrm{V}\mathrm{A}-0.11\bullet\:\mathrm{a}\mathrm{v}\mathrm{g}\mathrm{V}\mathrm{A}-0.07\bullet\:\mathrm{O}\mathrm{f}\mathrm{f}\mathrm{s}\mathrm{e}\mathrm{t}$$

Where $\:\varDelta\:$VA is the absolute visual acuity difference between the two eyes, while the avgVA is the average visual acuity at a specific defocus level. Since the simVA formula was developed using binocular data [11], in the current study, the obtained simVA was reduced by 7%, according to Campbell and Green [13].Then, the calculated binocular effect was applied for simulation of binocular defocus curves. To visualize binocular impression in mini-monovision, a quadratic summation of two USAF resolution test chart images with varying defocus offsets was performed [14, 15].

### Data analysis

We performed the data and image analysis using custom-made software developed in MATLAB (MathWorks, Natick, MA, USA).

## Results

### Power measurements

The measured refractive power of the IOLs is presented in Table 1. In all the lenses, we found a good agreement between our measured power and that provided by the manufacturer on the IOL label (i.e., nominal power).

**Table 1:** Measured refractive powers of the studied IOL samples

IOL Model	Acunex Quantum			RayOne EMV		
Label power [D]	10	20	30	10	20	30
Mean measured power [D]	9.81	20.11	30.03	10.06	20.27	30.38
SD of the measured power [D]	0.01	0.04	0.00	0.01	0.08	0.07

*IOL* intraocular lens, *SD *standard deviation

### Optical-quality assessment

**Fig. 1:**
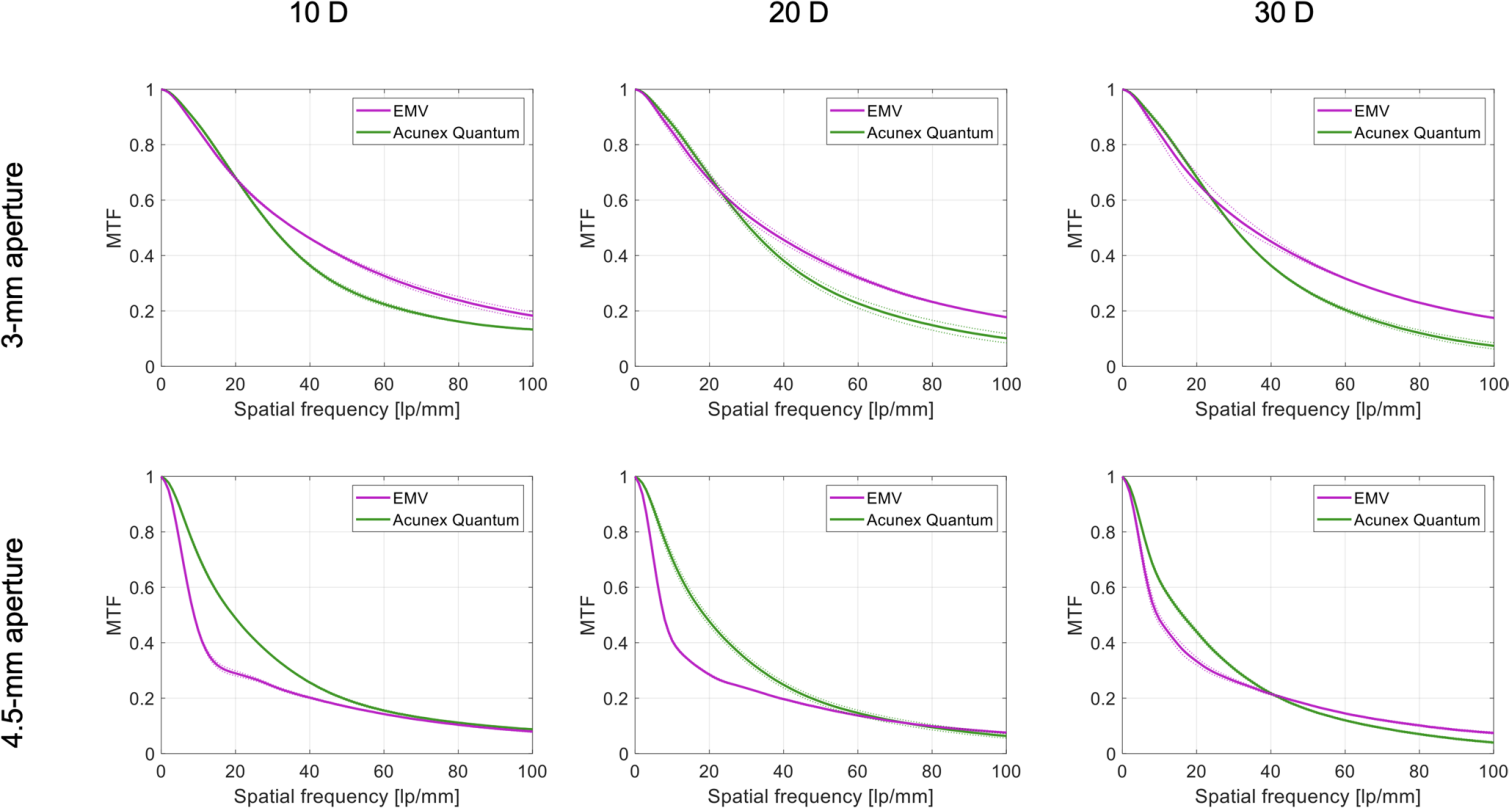
Modulation transfer function of the studied lenses at the best focus for 3- and 4.5-mm apertures. Dotted lines show the values of each lens separately; the solid lines refer to the average of two samples

The MTF curves measured at the best focus through the 3- and 4.5-mm apertures are presented in Fig. 1. At 3 mm, the RayOne EMV IOL demonstrated slightly higher MTF levels than the Acunex Quantum at spatial frequencies above approximately 25 lp/mm. The effect was consistent among the studied powers. Both models exhibited reduced MTF performance at 4.5 mm compared to the 3-mm aperture. The Acunex Quantum presented slightly higher MTF values up to 40 lp/mm in 10D samples and up to 60 lp/mm in 20D and 30D IOL samples.

**Fig. 2:**
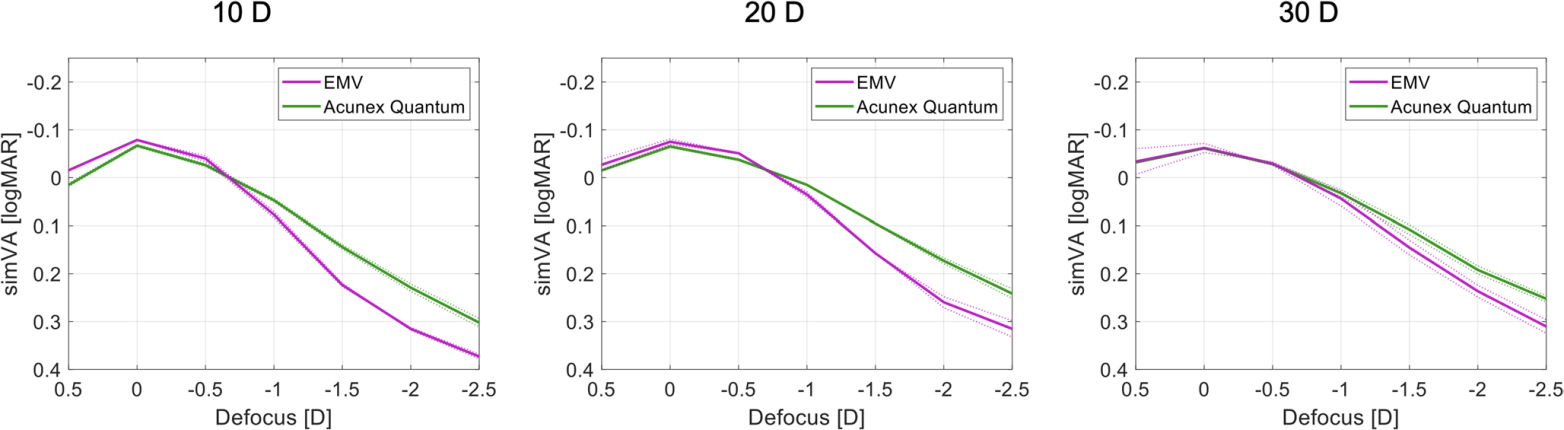
Simulated binocular visual acuity (plano implantation) as a function of defocus in IOLs with 10 D, 20 D, and 30 D power. Dotted lines show the values of each lens separately; the solid lines refer to the average of two IOLs

The binocular simVA derived from the multi-frequency wOTF^b^ metric at the 3-mm aperture is provided in Fig. 2. At the best focus, the difference between the two models was less than 0.02 logMAR. The depth of focus increased in both models with increasing IOL power. Compared to the RayOne EMV, the Acunex Quantum produced a more extended depth of focus at 10D and 20D, while increasing the power to 30D led to narrowing the gap between the two models. At a simVA cut-off of 0.2 logMAR, the Acunex Quantum provided a higher tolerance to defocus by approximately 0.4D in 10D IOL samples, 0.5D in 20D IOLs, and 0.3D in 30D IOLs.

### USAF resolution test chart

**Fig. 3:**
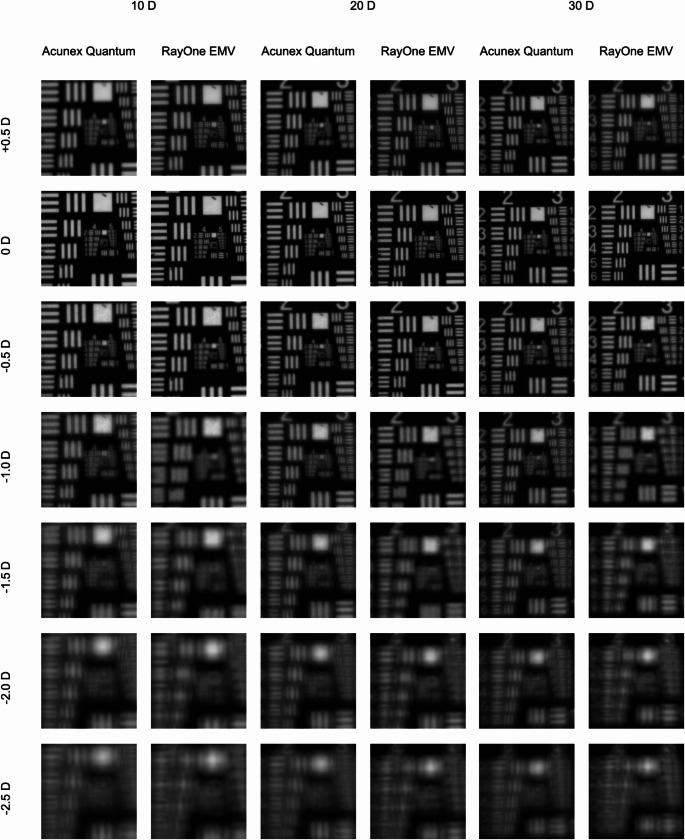
USAF-resolution targets recorded at a defocus range of + 0.5D to −2.5D and the 3-mm aperture

A visual assessment of the USAF resolution-test images in Fig. 3 confirms the numerical outcomes. While the RayOne EMV yielded images of slightly higher quality at a defocus of + 0.5D, both IOL models produced nearly identical results at no defocus. The Acunex Quantum demonstrated better resolution at defocus levels of −1.0D, −1.5D, and − 2.0D compared to the RayOne EMV.

### Simulation of binocular visual acuity in mini-monovision

**Fig. 4:**
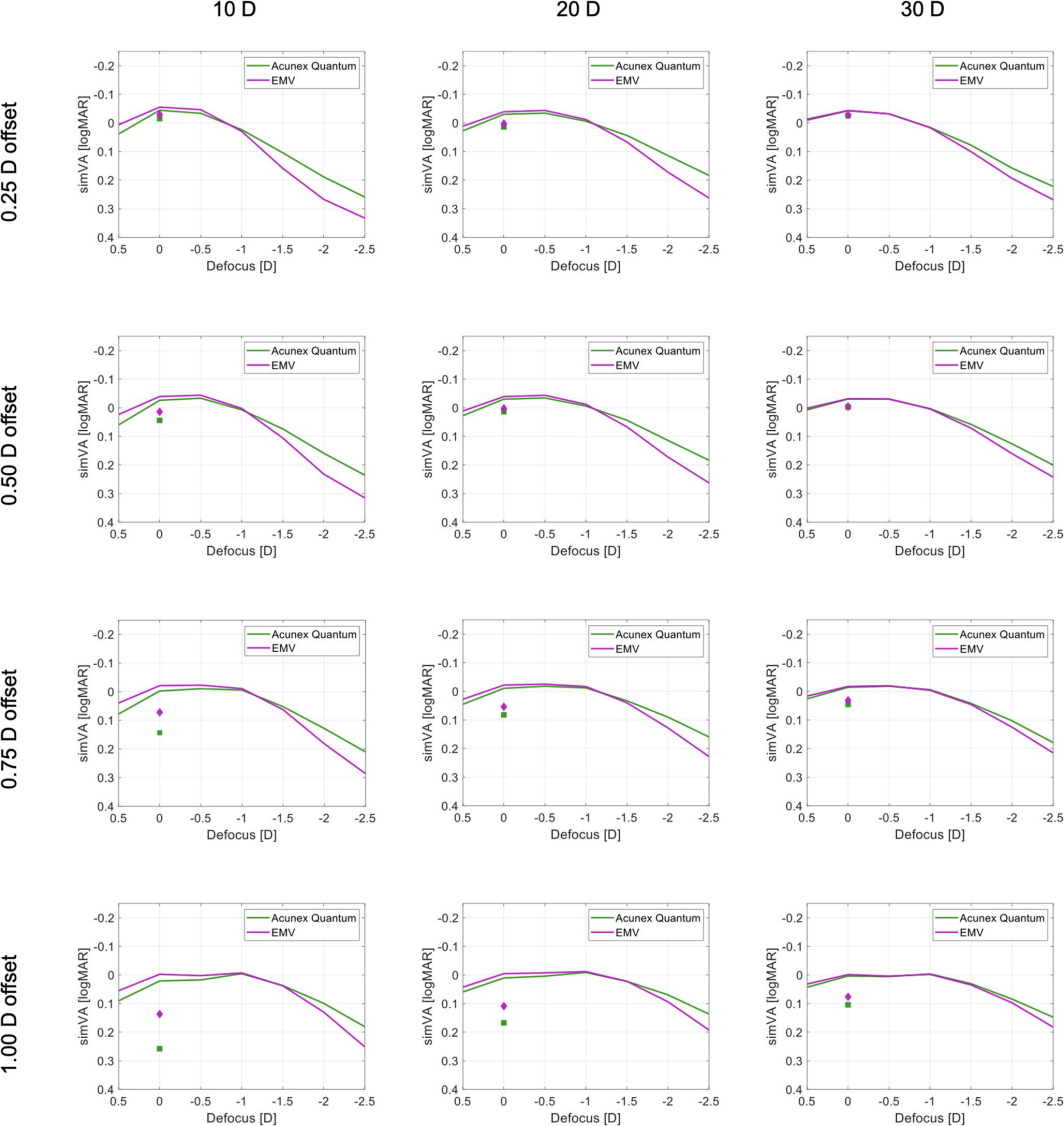
Simulated binocular defocus curves of the studied models with mini-monovision (solid lines). The filled squares and diamonds indicate monocular distance VA of a non-dominant eye with the Acunex Quantum and RayOne EMV, respectively

The effects of mini-monovision are presented in Fig. 4. The − 0.25D offset slightly widened the binocular depth of focus with a minimal reduction in simVA between the two refractive targets. The defocus tolerance was marginally improved at −2.0D for the two models, compared to plano binocular implantation. For the − 0.50D target, the simVA improvement in mini-monovision at −2.0D was on average 0.06 logMAR vs. binocular-plano implantation. The − 0.75D offset led an improvement of 0.09 logMAR at −2.0D with the Acunex Quantum. Likewise, a 0.13 logMAR improvement was observed with the RayOne EMV. The binocular simVA at −2.0D improved the most with the − 1.0D offset (approximately 0.11 logMAR, on average, with Acunex Quantum and 0.16 logMAR with RayOne EMV, compared to bilateral plano). The − 1.0D myopic offset provided the widest depth of focus, with a simVA of 0.1 logMAR and better in the range up to approximately − 2.0D to −2.4D with Acunex Quantum and − 1.8D to −2.1D with RayOne EMV. However, the distance simVA of the eye with the myopic target was reduced with the Acunex Quantum vs. EMV (0.26 vs. 0.14 logMAR) in the 10D comparison; for 20D, it was 0.17 vs. 0.11 logMAR, and for 30D, it was 0.10 vs. 0.08 logMAR. Compared to a binocular-plano implantation, the − 1.0D offset reduced the binocular simVA at far distance by approximately 0.06 to 0.08 logMAR.

### Simulation of binocular visual impression in mini-monovision

**Fig. 5:**
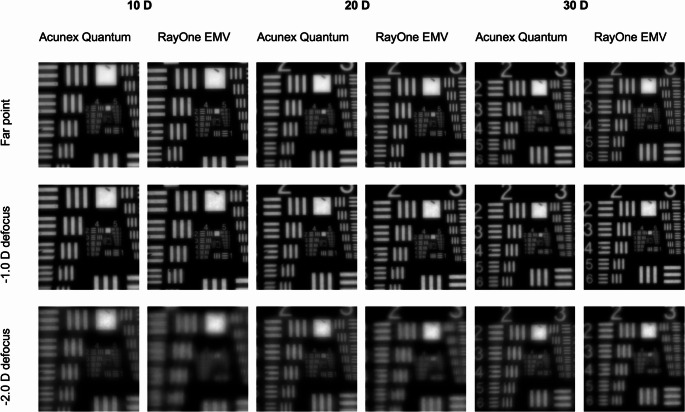
Simulated binocular USAF-resolution targets recorded at 0 D, −1.0 D, and − 2.0 D of defocus, created using the quadratic summation of two images with the myopic offset of −1D

Figure 5 presents the quadratic summation of images with the − 1.0D offset, representing the condition where distance vision is particularly susceptible to being compromised. The visual inspection of the simulated binocular viewing reflects the conclusions of the numerical analysis presented in Fig. 4. The image analysis predicts a low, albeit noticeable, negative impact of the − 1.0D myopic offset on binocular distance-vision, less noticeable with the RayOne EMV, and a marked improvement of intermediate vision, especially with the Acunex Quantum. Supplementary Fig. 1shows the comparison between the USAF-chart photographs taken with the simulated − 0.25D, − 0.50D, -0.75D and -1.00D offsets for a 20D lens.

## Discussion

Recently, manufacturers have introduced monofocal IOL models with enhanced intermediate function which do not meet the requirements set by the American Academy of Ophthalmology for extended-depth-of-focus (EDoF) lenses, and these are therefore termed enhanced monofocal or mono-EDoF IOLs [5, 16, 17]. They typically utilize higher-order aberrations to increase the depth of focus [5, 18]. One of the earlier examples of this group of IOLs is the Tecnis Eyhance (Johnson & Johnson Vision, Jacksonville, FL, USA), which features gradually increasing power from the periphery to the center of the IOL optic [18]. Another example of enhanced monofocal IOLs is the Isopure 1.2.3. (PhysIOL, Liège, Belgium), which also features a higher power at the center of the lens, resulting in high asphericity [19]. The IOLs evaluated in this study, RayOne EMV and Acunex Quantum, are also positioned in the market as enhanced monofocal IOLs. Similar to the previously mentioned ICB00 and Isopure lenses, they also feature monofocal optical designs that utilize asphericity to increase the depth of focus. Limited clinical data is available on the RayOne EMV and the Acunex Quantum lenses. In a non-comparative study, García-Bella et al. reported a mean (± SD) binocular CDVA of −0.07 ± 0.05 logMAR and DCIVA at 66 cm of 0.24 ± 0.09 logMAR [20]. Compared to the previously described lenses, the intermediate vision reported for the RayOne EMV was approximately one line worse [20]. This is in agreement with our previous laboratory study, in which we compared different enhanced monofocal IOL models and found the RayOne EMV provides a lower defocus tolerance when compared to Isopure and Eyhance [5]. Alarcon et al. also reported similar results with the RayOne EMV in their laboratory comparison of enhanced monofocal IOLs [21]. One study presented the clinical outcomes of the Acunex Quantum IOL, though it only reported distance visual acuity (CDVA: 0.01 ± 0.03 logMAR) [22]. A laboratory study by the same group found an increased focus depth with the Acunex Quantum IOL compared to the conventional monofocal Acunex AN6 IOL (Teleon Surgical, Spankeren, the Netherlands), which agrees with our findings [6].

In simulated plano-target implantation, the Acunex Quantum demonstrated a more extended intermediate range compared to the RayOne EMV. However, as the nominal power increased, a subsequent reduction in the depth of focus extension between the two lenses was observed, ranging from 0.3D to 0.5D. The smallest difference was observed for the 30D samples, attributed to enhancements in the defocus tolerance of the RayOne EMV with higher diopter powers. Since an increase in IOL power tends to lead to an increased SA, one may assume that this improvement can be explained by a surplus of SA produced by the RayOne EMV [23, 24]. The impact of SA on the depth of focus has been studied extensively, providing evidence that primary SA can effectively extend the visual range [25, 26]. The RayOne EMV’s simulated defocus curve resembled the performance of a monofocal lens in its defocus tolerance. Our earlier investigation indicated only minute differences between the RayOne EMV and a monofocal ZCB00 model for simulated emmetropia [5]. The current investigation suggests that the Acunex Quantum may provide a more extended range of vision than the RayOne EMV when considered for emmetropia.

Although enhanced monofocal IOLs were reported to provide a higher spectacle independence when compared to conventional monofocal IOLs, the improvement of the intermediate and near vision is generally considered insufficient to make these lenses suitable for patients asking for spectacle independence [25–27]. Mini-monovision aims to increase the depth of focus and spectacle independence when compared to a bilateral implantation with the emmetropic target [3]. The enhanced monofocal IOLs with this approach have a potential advantage against conventional monofocal lenses due to their already increased depth of focus. In addition, they should enable a larger myopic offset in the non-dominant eye without a significant decrease in binocular visual quality between the refractive targets of both eyes. Our analysis confirms that the investigated IOL models can be used in the mini-monovision configuration. A low offset of −0.25D or −0.50D appears to have a low impact on distance visual acuity, particularly in eyes with higher diopter power. With a higher offset, the negative effect on the vision of the eye with a myopic target is more noticeable. The RayOne EMV appears to provide a better distance vision in the myopic eye than the Acunex Quantum, especially at lower IOL powers, potentially resulting in a more similar visual impression in both eyes. These benefits diminish with higher powers due to the broadening of the Acunex Quantum defocus curve. The intermediate vision, however, appears to be better with the Acunex Quantum, as it offers a more extended intermediate range for each simulated offset.

Clinical data on the use of enhanced monofocal lenses in mini-monovision is scarce. Beltraminelli et al. compared mini-monovision outcomes in patients with the enhanced monofocal Eyhance IOL vs. conventional monofocal IOLs [28]. Although they reported superior outcomes at near and intermediate distances in enhanced monofocal IOL patients, the data is difficult to interpret as these patients also had more myopic postoperative spherical equivalents in both dominant and non-dominant eyes, and the targeted offset varied from − 0.5D to −1.25D [28]. Tomagova et al. presented the outcomes using the Isopure IOL with an offset of approximately − 0.5D and reported generally comparable results to the studies of this lens which targeted emmetropia in both eyes [29, 30]. The binocular uncorrected visual acuity was − 0.02 ± 0.07 logMAR at far, 0.13 ± 0.11 logMAR at intermediate, and 0.40 ± 0.20 logMAR at near distances, with 96%, 95%, and 34% of patients feeling comfortable with their vision without spectacles at far, intermediate, and near distances, respectively [29]. Unfortunately, the study did not report the power of IOLs implanted, which we found to influence the amount of the depth of focus. The lack of a clear difference between the reported outcomes with the mini-monovision approach in the study by Tomagova et al. and those targeting bilateral emmetropia could also have been due to a significant overlap in achieved refraction between the eyes with targeted emmetropia and those with a myopic target, due to limitations of the IOL power calculation accuracy [29]. The mean (± SD) achieved spherical equivalent was − 0.15 ± 0.41 D in the eyes with an emmetropic target and − 0.46 ± 0.35 D in those with a myopic target, indicating the effective difference between the eyes was less than the planned 0.5D [29]. Goldberg et al. used a larger offset of −0.75D to −1.75D with a conventional monofocal IOL and their patients achieved a high rate of spectacle independence, with only 9% requiring spectacles for reading [3]. Tan et al. used the − 0.5D offset with the Tecnis Symfony diffractive EDoF IOL [31]. They reported superior intermediate and near vision compared to the bilateral emmetropia using the same lens [31]. We could not find clinical studies that used the mini-monovision approach with RayOne EMV or the Acunex Quantum IOLs. Our laboratory data suggest that a mini-monovision approach can be successfully applied with both lenses, with the Acunex Quantum potentially providing a greater depth of focus irrespective of IOL power and the RayOne EMV a more similar visual impression far, particularly in eyes requiring lower-power IOLs.

Optical bench measurements of IOLs enable an objective comparison of different IOL models, and the optical quality metrics obtained correlate highly with clinical visual acuity [11, 32]. However, it should be noted that the methodology was developed to predict visual acuity in cases of a bilateral IOL implantation with identical refractive targets [11]. It is not clear if the amount of binocular summation, which is the improvement of vision in binocular viewing in comparison to monocular vision of the better-seeing eye, in mini-monovision patients, meaningfully differs from the amount observed in bilaterally-emmetropic patients, where it is usually √2 or higher [13, 33–35]. In conventional monovision, where the refraction difference between the eyes is higher, it has been suggested that the ratio of binocular summation could be closer to or even less than 1, meaning a deterioration of binocular vision when compared to monocular viewing [36, 37].

To address these limitations of optical bench measurements in predicting binocular performance, an alternative approach involves the use of the SimVis Gekko. This is a binocular visual simulator that enables direct assessment of visual performance under natural viewing conditions and enables the quantification of binocular summation, a key factor in mini-monovision. The system uses temporal multiplexing by incorporating tunable lenes for rapid changes of the optical power [38–40]. Notably, previous studies have shown strong correlations between SimVis based preoperative simulations and actual postoperative outcomes, supporting its reliability in predicting binocular visual outcomes [41, 42]. In our study, we used a formula developed from binocular vision simulations with the SimVis Gekko to predict the binocular effect of mini-monovision, accounting for varying binocular summation at different offsets.

Our visual acuity simulations assume that the postoperative outcomes are free of astigmatism. In clinical cases, however, small amounts of uncorrected astigmatism often occur, which should be considered a limitation of our study [43]. It remains the subject of future research to investigate visual acuity simulations in mini-monovision incorporating residual astigmatism, which may necessitate complex simulations as the impact on visual function depends not only on the amount of astigmatism but also on its type [44].

In conclusion, the Acunex Quantum performed better in extending the intermediate range on the optical bench than the RayOne EMV. The observed improvement varied, however, across IOL powers, suggesting potential contributions from spherical aberration. This mini-monovision simulation study indicated the suitability of both lenses for this approach. We found the Acunex Quantum to potentially provide a greater depth of focus and the RayOne EMV a better visual impression in distance vision, with diminishing differences between the models at higher powers.

## Supplementary Information

Below is the link to the electronic supplementary material.

Supplementary Material 1 (PNG 2.13 MB)

High Resolution Image (TIF 7.79 MB)
