# Supplementary figures and images for: Refining mini-monovision with monofocal plus intraocular lenses
Source: Graefes Arch Clin Exp Ophthalmol. 2025 Nov 8;264(1):159–69. doi: 10.1007/s00417-025-07013-2 (PMC12906528; doi:10.1007/s00417-025-07013-2)

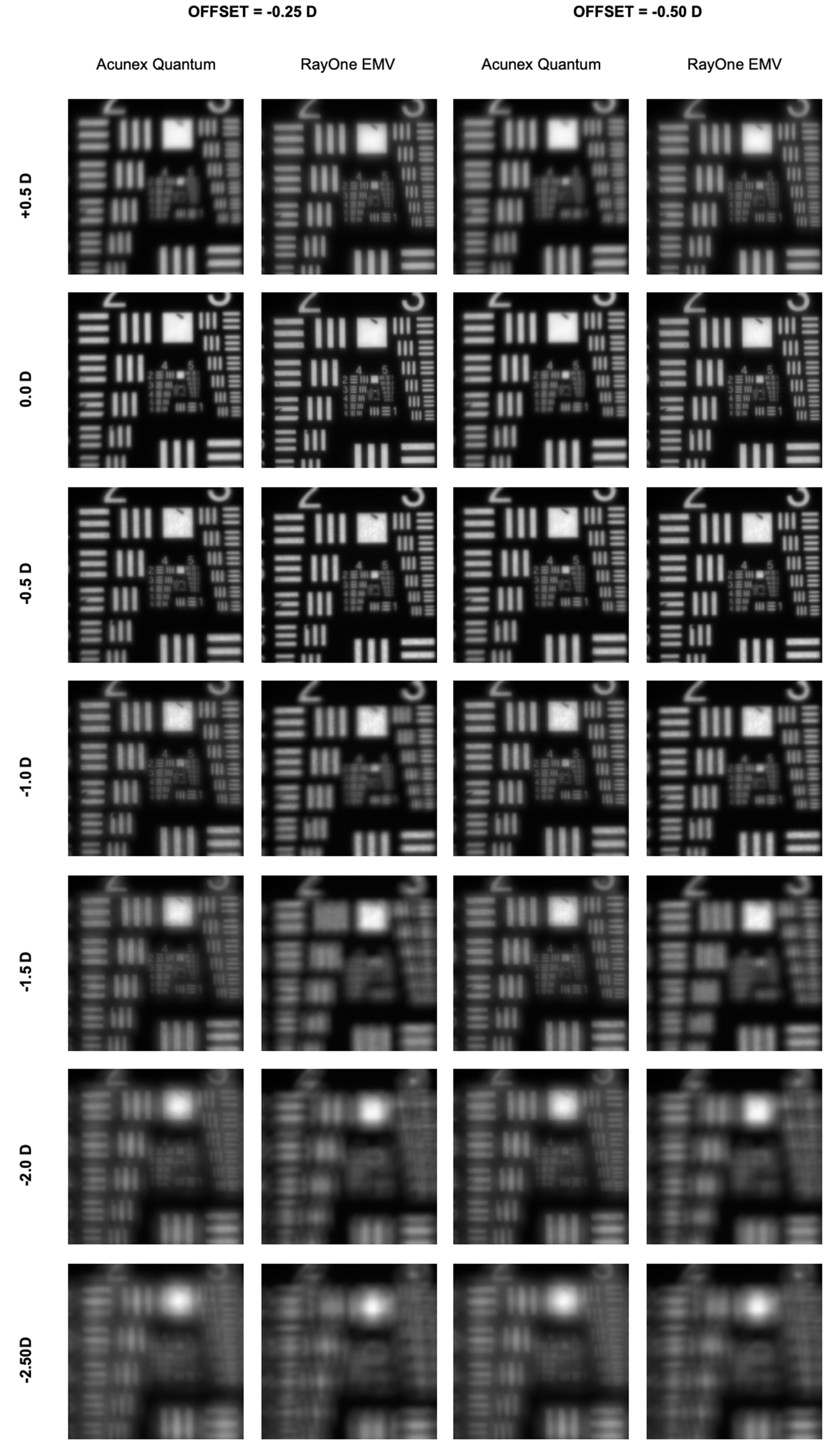

Supplement: Supplementary file 1 — Supplementary Material 1 (PNG 2.13 MB) [file 417_2025_7013_MOESM1_ESM.png]

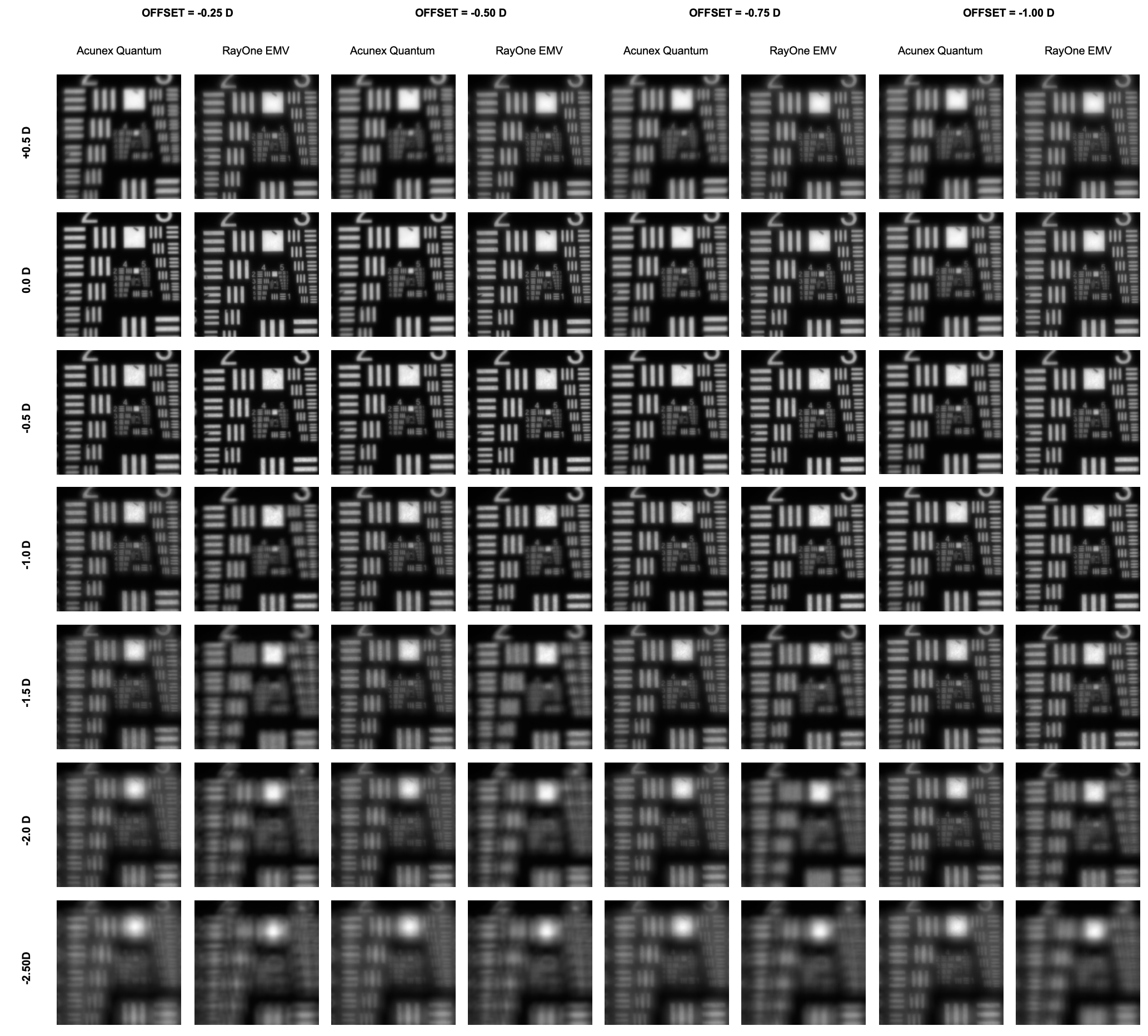

Supplement: Supplementary file 2 — High Resolution Image (TIF 7.79 MB) [file 417_2025_7013_MOESM2_ESM.tiff]
